# Bone mineral density and cytokine levels during interferon therapy in children with chronic hepatitis B: does interferon therapy prevent from osteoporosis?

**DOI:** 10.1186/1471-230X-5-30

**Published:** 2005-09-19

**Authors:** Ali Gur, Bünyamin Dikici, Kemal Nas, Mehmet Bosnak, Kenan Haspolat, Aysegul Jale Sarac

**Affiliations:** 1 Department of Physical Medicine and Rehabilitation, Medical Faculty, Dicle University, Diyarbakir – Turkey; 2 Department of Pediatrics, Medical Faculty, Dicle University, Diyarbakir – Turkey

## Abstract

**Background:**

Our aim was to determinate bone mineral density (BMD), levels of biochemical markers and cytokines in children with chronic hepatitis B treated with interferon (IFN)-alpha and to investigate effect of IFN-alpha therapy on these variables. To the best of our knowledge, this is first study carried out about BMD and cytokine levels in pediatric patients with chronic hepatitis B treated with IFN-alpha.

**Methods:**

BMD, levels of parathyroid hormone (PTH), osteocalcin, C-terminal cross-linking telopeptide of type I collagen (CTX), calcium, alkaline phosphates (ALP), cytokines as TNF-alpha, interleukin (IL)-1_β_, IL-2r, IL-6, and IL-8 were studied in 54 children with chronic hepatitis B (4–15 years old) treated with interferon alone (n = 19) or in combination with lamivudine (n = 35) for six months and as controls in 50 age-matched healthy children.

**Results:**

There was no significant difference in respect to serum IL-1_β_, TNF-α and osteocalcin levels while serum IL-2r (p = 0.002), IL-6 (p = 0.001), IL-8 (p = 0.013), PTH (p = 0.029), and CTX (p = 0.021) levels were higher in children with chronic hepatitis B than in healthy controls. BMD of femur neck (p = 0.012) and trochanter (p = 0.046) in patients were higher than in healthy controls. There was a statistically significant correlation between serum IL-1_β _and osteocalcin (r = -0.355, p < 0.01); between serum IL-8 and CTX levels (r = 0.372, p = 0.01), and ALP (r = 0.361, p = 0.01); between serum ALP and femur neck BMD (r = 0.303, p = 0.05), and trochanter BMD (r = 0.365, p = 0.01); between spine BMD and IL-2R (r = -0.330, p < 0.05).

**Conclusion:**

In conclusion, our study suggest that BMD of femur, serum IL-2r, IL-6, IL-8, PTH, and CTX levels were higher in children with chronic hepatitis B treated with IFN-alpha alone or combination with lamivudine than in healthy children. High femur BMD measurements found in patients may suggest that IFN-alpha therapy in children with chronic hepatitis B could contribute indirectly to prevent from hip osteoporosis. Additionally, further investigations on effects of IFN-alpha for bone structure in children should be performed in the future.

## Background

Bone development during childhood and adolescence is a key determinant of adult skeleton health. A reduced bone mass is associated with increased fracture risk in adults as well as in children. Peak bone mass, which is reached by early adulthood, serves as a bone reserve for the remainder of life, therefore childhood and adolescence are crucial periods for bone development. Strategies implemented for optimization of bone acquisition, as well as factors adversely affecting bone growth during these susceptible periods can have potentially long-standing consequences [1].

Recent studies have reported that osteodystrophy occurs not only in patients with alcoholic cirrhosis, but also in those with cirrhosis induced by hepatitis B and C viruses [2,3]. Because of improved treatment, patients with cirrhosis are living longer, an increasing proportion of such patients are found to have bone disease [4].

It is postulated that chronic liver disease and its complications might be responsible for activating some mediators [5,6]. It is further postulated that these mediators, such as some cytokines, might be the final common pathway leading to bone loss in parenchimal liver disorders [7].

A variety of compounds, including hormones and nutrients, are known to modulate bone remodelling. In addition, to these well-characterized substances, the immune system plays a role in this process through the involvement of pro-inflammatory cytokines [8]. Much interest has been focused on the role of the immune system in bone remodeling, and in particular, on the potential influence of cytokines upon the autocrine and paracrine regulation of bone cell activity [9-12]. Cytokines possess an important role in the regulation of bone resorption and formation during pathologic bone remodeling, and they also play a role during normal bone remodeling [13]. Significantly, IL-6 is a potent activator of ostoclasts and bone resorption. Similarly, other cytokines, such as IL-1, IL-11 and TNF influence ostoclast function and the age associated dysregulation of these cytokines may also contribute to the development of osteoporotic bone disease [8]. IL-8 is a chemokine of importance in inflammatory processes, and causes an increase in the levels of parathyroid hormone (PTH) mRNA. This suggest that IL-8 and inflammatory events may play a role in bone homeostasis by acting upon the parathyroid gland [14-16].

Interferon (IFN) has been shown to be effective in inducing inhibition of viral replication, normalization of liver tests and even improvement of liver histology in HBV-related liver diseases and it is known that IFN-alpha may affect bone turnover. There is limited information about the long-term effect of IFN-alpha therapy on bone metabolism.

A large number of studies on hepatic osteodystrophy in adult have reported recent advances in research on bone metabolism. However, bone metabolism in children has been regarded as differential diagnosis of bone resorption, pathological mechanisms and effects of IFN-alpha has not been elucidated.

Our aim was to determinate bone mineral density (BMD), levels of biochemical markers and cytokine in children with chronic hepatitis B treated with IFN-alpha and to investigate effect of IFN-alpha therapy on these variables. To the best of our knowledge, this is first study carried out about BMD and cytokine levels in pediatric patients with chronic hepatitis B treated with IFN-alpha. In view of the cost and widespread universal use of this drug in all age groups, especially with the epidemic of hepatitis B and C, we feel that such a detailed study is important.

## Methods

BMD, levels of PTH, osteocalcin, CTX, calcium, cytokines as TNF-alpha, interleukin (IL)-1_β_, IL-2r, IL-6, and IL-8 were studied in 54 children with chronic hepatitis B (4–15 years old) treated with interferon alone (n = 19) or in combination with lamivudine (n = 35) for six months and as controls in 50 sex and age-matched healthy children.

This study was performed in Dicle University, Diyarbakir, Turkey. Informed consent was taken from the parents of patients and sufficient information was given to them about the disease course and the treatment procedure at the beginning of the study. The study was approved by the local ethics committee.

BMD of the spine and hip (neck, trochanter) were measured by dual-energy x-ray absorptiometry (DEXA) (NORLAND, 6938CE, New York, USA). The variation coefficient for consecutive determinations on spine and femur images in our laboratory was 1.9% at the lumbar spine and 1.6 % at the femur region. All spinal scans were reviewed for evidence of vertebrae with collapse or focal sclerosis by an experienced radiologist.

The diagnostic criteria for chronic HBV infection were seropositivity for hepatitis B surface antigen (HBsAg), lack of anti-hepatitis B surface antibodies (anti-HBs), and presence of anticore IgG antibodies (anti-HBc). All patients had been infected with HBV for more than 2 yr. The mean time from the presumed onset of HBV infection, defined as at least from the first documented elevation of serum liver enzyme levels, to the study was 3.9 ± 3.2 yr. Knodell's histological activity index was used to evaluate necro-inflammation and fibrosis in biopsy samples from all patients. Mean inflammatory score was 5.1 ± 2.4, and the mean fibrosis score, 1.2 ± 1.1. None of all patients had cirrhosis.

The diagnosis of all patients was confirmed after a thorough laboratory investigation for their symptoms of icterus, abdominal pain, fatigue and loss of appetite. Moreover, family members of patients were tested for serologic parameters of HBV in order to determine possible vertical or horizontal transmission.

Patients were excluded from the study, after the screening, if they were more than 16 years old; if they had having positive test results for antibody to hepatitis D virus, hepatitis C virus, or human immunodeficiency virus; having decompensate liver disease (defined by a serum bilirubin level more than 2.5 times the upper limit of normal, a prothrombin time prolonged by more than 3 s and a serum albumin level lower than 3 g/dl or a history of ascites, variceal hemorrhage, or hepatic encephalopathy); if they have evidence of autoimmune hepatitis (defined as an anti-nuclear antibody titer higher than 1/160) or metabolic liver disease (Wilson's disease, hemochromatosis, deficit of α-1 antitrypsin); if they had received investigational drug within 30 days before enrollment. Patients were also excluded if they had a total white-blood-cell count less than 2500/m^3^, a neutrophil granulocyte count less than 1000/mm^3 ^and a value of haemoglobin less than 10 g/dl; if they were in poor clinical condition and/or had serious medical diseases (e.g. malnutrition, cardiomyopathies, diabetes, hypertension, neurologic, metabolic, autoimmune and neoplastic diseases).

19 patients with hepatitis B who entered the study received ten million units/ body surface area (max 10 million units) three times per week of recombinant interferon alpha 2b alone and 35 patients received interferon alpha 2b in same dosage in combination with lamivudine 4 mg/kg (max 100 mg) for six months. Recombinant interferon alpha-2b was administered subcutaneously by qualified medical staff or by the parents of patients after adequate training.

Blood samples were obtained after an over-night fast; precautions were taken to avoid contamination. Freshly drawn blood (15 ml) samples were obtained and immediately centrifuged at 200 × g (20 min at 24°C). For these tests HBV antigens and antibodies were assessed by qualitative micro-particle enzyme immunoassay (Organon Teknika BV, Boxtel, The Netherlands) HBV-DNA by Digene Hybride Capture Systems (Beltsville, MD 20705, USA). Serum levels of cytokines were determined using IMMULITE diagnostic kits (DPC-Diagnostic Products Corporation, USA). This diagnostic kit is an in vitro enzyme-linked immunosorbent assay for the quantitative measurement of human cytokines in serum. The serum Osteocalcin level was measured with a commercially available N-MID Osteocalcin Electrochemiluminescence Immunoassay kit (Roche Diagnostics GmbH, Mannheim, Germany). The serum levels of CTX were determined by Elecsys β-Crosslaps commercially available immunoassay kit (Roche Diagnostics GmbH, Mannheim, Germany). Serum PTH was measured by a two-site immunoradiometric assay using a commercially available kit (Nichols Institute). Serum and urinary chemical estimations were performed using Beckman-Synchron CX-5 technology.

### Statistical analysis

The data obtained were analyzed using the Statistical Package for the Social Sciences (SPSS 10.0). Results in patients with chronic hepatitis B and controls were compared using Student's unpaired t test. Results in patients treated with IFN-alpha alone and combination therapy were compared using Mann -Whitney -U test. Pearson's correlation test was used for correlation analysis. All statistical tests were 2-sided; p < 0.05 was considered to be statistically significant. Values are expressed as the mean ± standard deviation.

## Results

The mean age of patients and healthy control groups were 10.23 ± 3.12 and 10.02 ± 2.84 years, respectively. Patients group consisted of 43 females and 11 males and there were 39 females and 11 males in controls group. In both groups, mean body mass index and other demographic characteristics were similar. There was no statistically significant difference in any demographic characteristics between the groups (p > 0.05).

There was no statistically significant difference between patients received recombinant interferon alpha-2b alone and combination with lamivudine (Table 1).

Laboratory data of both groups are shown in Table 2. While serum IL-2r (p = 0.002), IL-6 (p = 0.001), IL-8 (p = 0.013), PTH (p = 0.029), and CTX (p = 0.021) levels were higher in children with chronic hepatitis B than in healthy controls, there was no significant different in respect to serum IL-1_β_, TNF-α and osteocalcin levels. BMD of femur neck (p = 0.012) and trochanter (p = 0.046) in patients were higher than in healthy controls.

Correlation between laboratory data and BMD measurements of patients group are shown in Table 3. There was a statistically significant correlation between serum IL-1_β _and osteocalcin (r = -0.355, p < 0.01), ALT (r = 0.494, p = 0.01), and AST (r = 0.528, p = 0.01); between serum IL-8 and CTX levels (r = 0.372, p = 0.01), and ALP (r = 0.361, p = 0.01); between serum ALP and femur neck BMD (r = 0.303, p = 0.05), and trochanter BMD (r = 0.365, p = 0.01); between spine BMD and IL-2R (r = -0.330, p < 0.05).

## Discussion

Osteoporosis is an otherwise healthy child or adolescent is rare, although cases of idiopathic osteoporosis have been described. Rather, pediatric osteoporosis is increasingly recognized in the setting of chronic illness related to the disease itself or its treatment. A large number of studies on primary osteoporosis have reported recent advances in research on bone metabolism. However, secondary osteoporosis has been regarded as a differential diagnosis of primary osteoporosis, and its pathological mechanisms have not been elucidated compared with those of primary osteoporosis [17].

Bone manifestations are well-known extrahepatic complications of chronic liver diseases [18,19]. In these patients, several factors contribute to the development of bone disease. In particular, malnutrition, immobilization, and hormonal changes are causes for deteriorating bone metabolism in patients with chronic liver diseases [19]. The mechanism leading to osteoporosis is still unclear. The equilibrium between bone formation and bone resorption is disturbed [20], and, apart from the decreased activity of osteoblasts [21], there are also studies indicating an increase in osteoclast activity [22]. In contrast to primary biliary cirrhosis and primary sclerosing cholangitis, no disease-specific association between chronic hepatitis B, C, and D virus infection and osteoporosis is documented. Only few studies on bone metabolism have been performed in patients suffering from chronic viral hepatitis, especially before and after liver transplantation [23-25].

Bone disease in patients with chronic active hepatitis is usually asymptomatic and is characterized by decreases in BMD. Histomorphometric analysis of bone biopsies from the iliac crest of patients with chronic active hepatitis shows osteoporosis with decreased trabecular bone volume and no osteomalacia. Bone remodeling is regulated by a number of growth factors, cytokines, systemic peptides and steroid hormones. Proinflammatory cytokines appear to have a role in the development of chronic liver disease. IL-1b and TNF-alpha are involved in liver fibrogenesis [26]. The activation of the cytokine cascade, induces fibroblast proliferation and parenchymal inflammatory response producing liver damage [26,27].

The prevalence of osteoporosis among patients with chronic liver diseases ranges from 10% to 60% [28-31], the highest being observed in cholestatic liver disease and alcoholic liver disease. A recent study revealed that the prevalence of osteoporosis in patients with cirrhosis secondary to hepatitis B or C was nearly 50% [32]. Most studies of bone disease were performed in patients with cirrhosis. Nevertheless, little is known about the occurrence of bone disease in non-cirrhotic patients with chronic hepatitis B or C [33].

Interleukins and lymphokines may play a role in the bone remodeling process [1]. The calcitonin-like effect of IFN-γ is difficult to interpret if one assumes that the immune interferon actually brings about the fusion of monocytes into osteoclasts. If this actually occurred in bone this would force the conclusion that these cells can not be activated in the presence of IFN-γ. This could also explain the reduced effectivity of PTH in the presence of the immune interferon. Although IL-1_β _and TNF-α may be involved in the bone remodeling process, we did not find any significant difference when we compared their serum levels in children with chronic hepatitis B and healthy controls. It should be borne in mind that several disputes exist among researches concerning cytokines and pathologic bone remodeling, especially concerning the secretion of cytokines into the peripheral blood. This may be related to effect of IFN-alpha therapy.

Since biochemical markers of bone turnover are important in the assessment of osteoblastic and osteoclastic functions, we measured the serum osteocalcin and C-terminal cross-linking telopeptide of type I collagen levels. Osteocalcin is a noncollagenous protein secreted by osteoblasts and is widely accepted as a marker for osteoblastic activity [34] and bone formation [35], whereas serum CTX, as a collagen-degradation product is a marker of bone resorption [36].

In some studies, it has been reported that the serum osteocalcin levels are higher in cirrhosis patients, which means that cirrhotic patients have high turnover osteoporosis [37]. However, some authors reported that the serum osteocalcin levels were lower in cirrhotic patients and the osteopenia in these patients was not due to a decrease in bone formation [38,39].

In hepatocellular dysfunction, some authors reported that the serum parathyroid hormone levels were higher [40], and others reported them as unchanged [29]. Another report showed that the increase in bone resorption might be the result of decreased PTH degradation [41]. In our study, while serum PTH and CTX levels were higher in children with chronic hepatitis B than in healthy controls, there was no significant different in respect to serum osteocalcin and ALP levels.

IFN-alpha has numerous clinical applications but is used most extensively in the treatment of chronic hepatitis B and chronic hepatitis C. Research into the effects of IFN-alpha on bone mineral metabolism has been very sparse, and the majority of studies reflect in vitro models. The exact mechanism of positive effect on bone mineral metabolism by IFN-alpha is not completely understood although a number have been postulated. Both in vivo and in vitro studies demonstrate that IFN-alpha decreases bone resorption, whereas osteoblast may or may not be affected in vivo [42]. An in vitro study on the effects of IFN-alpha on human bone marrow stromal cells showed that IFN-alpha decreased the production of IL-1b [43], which has been shown to stimulate osteoclastic bone resorption [44].

Takayanagi et al. [45] reported that there is cross-talk between the tumour necrosis factor and IFN families of cytokines, through which IFN-gamma provides a negative link between T-cell activation and bone resorption. Authors stated that the findings of their study may offer a therapeutic approach to treat the inflammation-induced tissue breakdown. IFN-alpha clearly decreases bone resorption, but in vitro data suggest that there is decreased formation with increased differentiation of osteoblasts, whereas the in vivo work suggests that osteoblasts are not suppressed by IFN-alpha [42]. Thus, IFN-alpha could be increased BMD in children with chronic hepatitis by one or a combination of these mechanisms. In our study, because there is no statistically significant difference between patients received IFN-alpha alone and combination therapy we think that changes in BMD biochemical markers and cytokines are related to IFN-alpha treatment.

Solis-Herruzo et al. [46] reported that adult male patients receiving ribavirin and IFN-alpha had a lower bone mass than those receiving IFN-alpha only; this suggests that ribavirin was responsible for the decrease in bone mineral density. This was, however, a cross-sectional study and did not evaluate patients before treatment, possibly leading to inconsistent conclusions. Trombetti et al. [47], on the other hand, did not find any effect of ribavirin in bone metabolism. The impact, therefore, of IFN-alpha and ribavirin in bone remains unclear [48]. In our study, there was no statistically significant difference between patients with hepatitis B received IFN-alpha alone and combination with lamivudine.

In our study, BMD values of femur, but was not spine, in patients were higher than in healthy controls. High BMD values of femur postulated that IFN-alpha therapy might be responsible for inhibiting some mediators. It is further postulated that these mediators, such as some cytokines, might be the final common pathway leading to bone loss in parenchimal liver disorders. Because interferon inhibits the formation of osteoclast-like cells [49], interferon treatment may increase BMD. However, interferon is expensive and is thus inappropriate for the treatment of bone lesions. But, it is postulated that in patients treated with IFN-alpha may not need additional therapy for the treatment of bone resorption.

## Conclusion

In conclusion, our study suggest that BMD of femur, serum IL-2r, IL-6, IL-8, PTH, and CTX levels were higher in children with chronic hepatitis B treated with IFN-alpha alone or combination with lamivudine than in healthy children. High femur BMD measurements found in patients may suggest that IFN-alpha therapy in children with chronic hepatitis B could contribute indirectly to prevent from hip osteoporosis. Additionally, further investigations on effects of IFN-alpha for bone structure in children should be performed in the future.

## Abbreviations

BMD, bone mineral density; IFN, interferon; PTH, parathyroid hormone; ALP, alkaline phosphates; CTX, C-terminal cross-linking telopeptide of type I collagen; IL, interleukin; DEXA, dual-energy x-ray absorptiometry; HBsAg, hepatitis B surface antigen; Anti-HBc, anticore IgG antibodies; HBV, Hepatitis B virus

## Competing interests

The author(s) declare that they have no competing interests.

## Authors' contributions

AG participated in the design of the study and performed the statistical analyses.

BD participated in the design of the study and screened of subjects.

KH and AJS conceived of the study, and participated in its design and coordination.

KN and MB participated in the sequence alignment.

All authors read and approved the final manuscript.

## Pre-publication history

The pre-publication history for this paper can be accessed here:



## Figures and Tables

**Table 1:** Comparisons of clinical features in patients with chronic hepatitis B treated with IFN alone or IFN plus lamivudine

**Variables**	**IFN-alpha (n = 19)**	**IFN plus Lamivudine (n = 35)**	**P**
**TNF-α**	24.48 ± 8.46	26.60 ± 7.43	NS
**IL-1β**	7.56 ± 10.86	7.08 ± 12.47	NS
**IL-2r**	1472.42480.47	1333.72 ± 472.51	NS
**IL-6**	24.85 ± 31.46	25.57 ± 29.43	NS
**IL-8**	18.19 ± 13.88	18.73 ± 14.27	NS
**PTH**	42.03 ± 29.41	40.99 ± 26.92	NS
**s-CTX**	1.88 ± 0.68	1.96 ± 0.75	NS
**Osteocalcin**	65.49 ± 34.45	63.85 ± 32.67	NS
**Serum calcium**	9.28 ± 1.76	9.86 ± 1.69	NS
**ALP**	249.45 ± 71.34	193.09 ± 72.88	NS
**ALT**	62.83 ± 76.39	61.45 ± 81.54	NS
**AST**	59.16 ± 61.48	57.66 ± 59.82	NS
**L2–4 BMD**	0.63 ± 0.09	0.61 ± 0.14	NS
**Femur neck BMD**	0.78 ± 0.12	0.82 ± 0.17	NS
**Trochanter BMD**	0.67 ± 0.16	0.69 ± 0.12	NS

**Table 2:** Comparisons of laboratory data and BMD measurements of children with chronic hepatitis B treated with IFN-alpha alone or combination with lamivudine and healthy controls.

**Variables**	**Patients (n = 54)**	**Control (n = 50)**	**P**
**TNF-α**	25.54 ± 8.30	15.47 ± 6.77	NS
**IL-1β**	7.32 ± 12.13	5.48 ± 6.14	NS
**IL-2r**	1403.07 ± 455.67	1120.48 ± 492.37	**0.002**
**IL-6**	25.21 ± 39.27	5.98 ± 6.49	**0.001**
**IL-8**	18.46 ± 14.77	12.55 ± 7.36	**0.013**
**PTH**	41.51 ± 28.80	33.08 ± 18.22	**0.029**
**s-CTX**	1.92 ± 0.76	1.61 ± 0.52	**0.021**
**Osteocalcin**	64.67 ± 30.63	75.81 ± 31.10	NS
**Serum calcium**	9.57 ± 1.83	9.49 ± 1.52	NS
**ALP**	221.27 ± 67.27	219.66 ± 78.74	NS
**ALT**	62.14 ± 88.31	27.01 ± 42.41	**0.009**
**AST**	58.41 ± 65.57	31.72 ± 29.69	**0.007**
**L2–4 BMD**	0.62 ± 0.13	0.59 ± 0.12	NS
**Femur neck BMD**	0.80 ± 0.16	0.72 ± 0.15	**0.012**
**Trochanter BMD**	0.68 ± 0.14	0.62 ± 0.14	**0.046**

**Table 3:** Correlation between BMD and serum cytokines, and biochemical markers in children with chronic hepatitis C treated with IFN-alpha.

**IL-1β**	.501**												
**IL-2r**	.075	.096											
**IL-6**	.167	-.101	-.163										
**IL-8**	.137	.251	.248	-.126									
**PTH**	.005	-.012	.009	.121	.088								
**s-CTX**	-.087	-.253	.140	-.060	.372**	-.155							
**ALP**	-.013	-.049	-.055	-.092	.361**	-.102	.179						
**Osteocalcin**	.105	-.355**	.016	.135	-.205	-.101	.256	-.006					
**ALT**	.189	.494**	.087	-.161	.243	.122	-.257	.251	-.206				
**AST**	.214	.528**	.125	-.192	.253	.089	-.222	.252	-.155	.960**			
**L_2–4 _BMD**	-.159	.019	-.330*	.036	.138	.226	.122	.072	-.118	.122	.084		
**Femur Neck BMD**	-.112	-.043	-.246	.025	.105	.179	.067	.303*	-.034	.163	.097	.704**	
**Trochanter BMD**	-.111	-.039	-.237	-.006	.123	.090	.017	.365**	-.125	.198	.125	.697**	.943**
	**TNF-α**	**IL-1β**	**IL-2r**	**IL-6**	**IL-8**	**PTH**	**s-CTX**	**ALP**	**Osteocalcin**	**ALT**	**AST**	**L_2–4 _BMD**	**Femur Neck BMD**

** Correlation is sigificant at the 0.01 level (2-tailed).

* Correlation is significant at the 0.05 level (2-tailed).
